# Role of Inflammatory Cell Subtypes in Heart Failure

**DOI:** 10.1155/2019/2164017

**Published:** 2019-09-02

**Authors:** Derek Strassheim, Edward C. Dempsey, Evgenia Gerasimovskaya, Kurt Stenmark, Vijaya Karoor

**Affiliations:** ^1^Cardiovascular Pulmonary Research Laboratory, University of Colorado Denver, Aurora CO, USA; ^2^Division of Pulmonary Sciences and Critical Care Medicine, University of Colorado Denver, Aurora CO, USA; ^3^Rocky Mountain Regional VA Medical Center, Aurora CO, USA; ^4^Department of Pediatrics, University of Colorado Denver, Aurora CO, USA

## Abstract

Inflammation is a well-known feature of heart failure. Studies have shown that while some inflammation is required for repair during injury and is protective, prolonged inflammation leads to myocardial remodeling and apoptosis of cardiac myocytes. Various types of immune cells are implicated in myocardial inflammation and include neutrophils, macrophages, eosinophils, mast cells, natural killer cells, T cells, and B cells. Recent clinical trials have targeted inflammatory cascades as therapy for heart failure with limited success. A better understanding of the temporal course of the infiltration of the different immune cells and their contribution to the inflammatory process may improve the success for therapy. This brief review outlines the major cell types involved in heart failure, and some of their actions are summarized in the supplementary figure.

## 1. Introduction

Heart failure develops secondary to left ventricular (LV) systolic and diastolic dysfunction. Systolic heart failure is also known as heart failure with reduced ejection fraction (HFrEF) and occurs when a decreased force of contractility of the left ventricle reduces the pumping capacity. Diastolic dysfunction, also known as heart failure with preserved ejection fraction (HFpEF), occurs when the muscle in the left ventricle becomes stiff, due to either change in contractile proteins of cardiac myocytes or effect of fibrosis on relaxation [1–4]. Right heart failure occurs in association with lung disease leading to pulmonary hypertension and as an end stage of left ventricular failure [5]. Risk factors that contribute to the development of heart failure include ischemic injury, hypertension, and metabolic syndrome and age [6]. Genetic cardiomyopathies occur in patients with autosomal dominant mutations in various sarcomeric proteins [7, 8]. Mechanical dysfunction, due to valve dysfunction, and aortic stenosis in the elderly cause pressure overload leading to cardiac hypertrophy and can progress into LV dysfunction [8]. Immune-based cardiomyopathies occur in autoimmune diseases and due to infectious agents (viral and bacterial) when the innate and adaptive immune systems are activated to coordinate a primary response [9].

The role of elevated inflammatory biomarkers in chronic HF and in disease progression is not clear [6]. Measurement of biomarkers in patients with systolic HF (either ischemic or nonischemic) and animal studies has demonstrated an elevation in proinflammatory cytokines (such as TNF-*α*, IL-1, IL-6, galectin 3, TNF receptor 1, and TNF receptor 2), during HF progression supporting the hypothesis that inflammation may contribute to HF.

Studies show that resident and recruited immune cells play a role in cardiac injury and are present in cardiac tissue early in disease [1–5, 9]. Initially, resident and infiltrating immune cells activate inflammatory/reparative pathways, and the relative balance between pathological inflammatory pathways and tissue reparative processes (physiological inflammation) defines the course of HF development [10]. Immune cells coordinate cardiomyocyte and noncardiomyocyte responses during maladaptive remodeling. These cells modulate not only cardiomyocyte function but also injury responses involving scar formation and interstitial fibrosis, which affect cardiac function. Therefore, modulating immune responses may be beneficial for therapy. In this review, we will discuss the role of different immune cells in noninfectious heart failure where activation of the immune system was a secondary response to dysfunction.

## 2. Immune Cells in the Heart

Immune cells identified in the heart either reside or infiltrate heart tissue and include macrophages, mast cells, monocytes, neutrophils, eosinophils, B cells, and T cells [11]. Inflammation in non-immune-mediated cardiac injury leads to secretion of inflammatory cytokines and chemokines such as IL-1, IL-6, TNF-*α*, and CC-chemokine ligand 2 (CCL2) and GM-CSF [11, 12]. An increase in levels of cytokines and chemokines leads to the recruitment of neutrophils and monocytes, from hematopoietic stem and progenitor cells (HSPCs) to the heart that scavenge dead cells [13]. Initially, the immune cells infiltrated the cells to scavenge dead and dying cardiomyocytes by digesting the tissue with proteolytic enzymes [14]. The inflammatory cascade is amplified by the dead cells causing further release of inflammatory cytokines which further amplify inflammation through their effects on leukocytes, endothelial cells, and cardiomyocytes [15]. Therefore, it is essential to identify the cytokines released by various cells and their role in the inflammatory cascade.

### 2.1. Neutrophils

Many studies show the involvement of neutrophils in the progression of cardiovascular diseases (CVD) including atherosclerosis, thrombosis, and ACS3 [16]. In ischemic cardiomyopathy, neutrophils infiltrate the infarcted myocardium and mediate tissue damage [17]. Neutrophils are short-lived and undergo apoptosis and shed the IL-6 receptor, which modulates the outcome of the inflammatory response by activating endothelial cells to recruit additional and a wider variety of leukocytes [14, 18–20]. As regulators of both innate and adaptive immune responses, neutrophils can influence chronic immune response and affect the function of dendritic cells as well as lymphocytes. Neutrophils have shown to improve cardiac healing following myocardial infarction (MI), by promoting macrophage polarization towards a reparative phenotype through the release of neutrophil gelatinase-associated lipocalin [21, 22]. Depletion of neutrophils does not affect infarct size, but worsens cardiac function and heart failure, and increases cardiac fibrosis [21]. In a rat model, histology and staining for myeloperoxidase (MPO) activity revealed a significant accumulation of neutrophils in *α*-toxin A-challenged hearts concomitant with reduced cardiac contractility and endothelial dysfunction [23]. Plasma levels of MPO correlated with LVEF and LV end-diastolic volume in a model system of HF [24]. Atherosclerosis studies have shown hyperlipidemia-associated neutrophilia and a role for neutrophils in plaque destabilization [25]. Neutrophil blood count has shown to correlate with the severity of coronary damage in patients with coronary artery disease [26]. Perivascular accumulation of neutrophils and macrophages has been observed in murine lungs in association with hypoxic pulmonary hypertension (PH) and monocrotaline-induced PH in rats [27]. In right ventricular (RV) failure associated with experimental pulmonary hypertension, increased expression of IL-1*β*, IL-6, and IL-10, accompanied by infiltration of both neutrophils and macrophages, is observed [18]. Although the role of neutrophils in the pathogenesis of PAH has not been studied, an increase in a neutrophil-to-lymphocyte ratio is observed in PAH patients [28]. An increase in neutrophil elastase causes tissue damage. In clinical studies, a neutrophil-to-leukocyte ratio (NLR) showed a strong association with HF and death [29, 30]. NLR is shown to be associated with chronic kidney disease, major cardiovascular events, and hospitalizations for HF in elderly patients [20]. NLR was an independent predictor of outcome in patients with stable coronary artery disease (CAD) and a predictor of short- and long-term mortality in patients with acute coronary syndromes (ACS), ST-elevation myocardial infarction (STEMI), and cardiac transplantation [31–33]. Despite the prominent role of neutrophils in inducing the chronic inflammatory response in the pathogenesis of many diseases, neutrophil-targeted treatments are still not available [34].

### 2.2. Macrophages

In the myocardium, different subsets of macrophages with different functions and origins have been identified, both protective and pathogenic [35]. Resident macrophages transition to a more reparative phenotype by dampening IL-6, TNF-*α*, and matrix metalloproteinase 9 (MMP9) expression and via extrinsic signals such as IL-10, which is produced by regulatory T (Treg) cells [11]. The consequence of this transition is the appearance of cardiac macrophages that produce TGF-*β* and VEGF thus promoting fibrosis and angiogenesis together with other factors such as myeloid-derived growth factor [22, 36]. Macrophages form 10% of noncardiomyocytes and maintain homeostasis by removing dying senescent cells and promoting angiogenesis [37–39]. In response to injury, the release of cytokines and chemokines recruits monocytes which differentiate into Ly6C high macrophages characterized as an M1 phenotype and play an essential role in clearing dead cells by phagocytosis and regulating proinflammatory signals [40]. A decrease in neutrophils and the appearance of Ly6C low macrophages with decreased production of inflammatory cytokines marks the transition from inflammation to repair [41]. In a mouse model of myocardial infarction, a decrease in CSF-1R inhibition in M2 macrophages was associated with a loss in left ventricular contractile function, infarct enlargement, decreased collagen staining, and increased inflammatory cell infiltration into the infarct zone [41]. Clodronate liposome depletion of macrophages following infarction in mice increased mortality and impaired cardiac repair [42, 43]. A recent study showed that CCR2 monocyte-derived macrophages infiltrate the heart early following pressure overload-induced hemodynamic stress and that this macrophage population is responsible for the activation of T cells and transition to failure. Blocking this response either pharmacologically or with antibody-mediated CCR2 depletion protects the heart from pathological left ventricular remodeling and dysfunction, T cell expansion, and cardiac fibrosis [35]. In mice with macrophage-specific deletion of IL-10, there is an improvement in diastolic function. IL-10 may promote fibrosis, by activating fibroblasts, increasing collagen deposition, and impairing myocardial relaxation [43]. In HFpEF mouse models, inflammation is influential in promoting cardiac fibrosis [44, 45]. In coronary artery disease, resident macrophages that are different from monocyte-derived macrophages contribute to pathology [46].

In atherosclerosis, bone marrow- and spleen-derived macrophages are major contributors only at the early disease stage, and resident macrophages become dominant at later phases of the disease. Macrophage subtypes with different functions are identified in the development and progression of atherosclerotic lesions. Macrophage accumulation in human plaques is linked with lesion progression and destabilization as well as with symptomatic coronary artery disease. Numbers of circulating monocytes increase with atherosclerosis and predict clinical outcome [47]. Mox phenotype differentiation is stimulated by the exposure to oxidized phospholipids and by high-level expression of heme oxygenase-1 (HO-1), through the activation of nuclear factor (erythroid-derived-2)-like 2 (NEF2L2) transcription factor [48–51]. In atherosclerotic plaques, macrophages adopt their phenotype under the influence of the degree of accumulated lipids and the production of specific mediators and immune factors and are responsible for the transition from a stable to an unstable plaque phenotype [52]. Mechanisms underlying the differentiation of macrophages by modulating the polarization of subpopulations would help to develop novel approaches aiming at slowing down the progression of atherosclerotic disease [53].

Macrophage accumulation in human plaques is linked with lesion progression and destabilization as well as with symptomatic coronary artery disease (CAD) [54]. Numbers of circulating monocytes is seen to be associated with atherosclerosis and to predict clinical outcome [33]. Increased numbers of macrophages were observed in myocardial biopsies from HFpEF patients and contribute to pathophysiology [43, 55]. Subsets of monocytes with different functions are identified in human patients [56]. Classical monocytes, Mon1, preferentially express cytokines IL-1*β*, IL-6, and MCP-1, intermediate monocytes, Mon2, produce anti-inflammatory IL-10, and nonclassical monocytes, Mon3, stimulate cytokine production in response to viral rather than bacterial load. Mon2 is shown to increase in heart failure and correlates with NYHA [57].

Macrophages have been implicated in the pathogenesis of experimental and human pulmonary hypertension [27]. Clodronate liposome depletion of macrophages prevented hypoxia-induced vascular remodeling. An increase in perivascular macrophages in vascular lesions of human patients with PH is observed [46, 47]. Notably, reduced right ventricle systolic pressure, right ventricle hypertrophy, and pulmonary vascular remodeling were noted in CX3CR12/2 mice, but not in CCL22/2 or CX3CR12/2/CCL22/2 mice, compared with wild-type mice [58]. In IPAH, CCL2 is thought to contribute to the inflammation process. Manipulating macrophages in both inflammatory diseases have been demonstrated to be beneficial in improving the outcome in preclinical mouse models, holding promise for the future design of therapeutic interventions [59].

### 2.3. Natural Killer Cells

NK cells comprise the most substantial subset of the innate lymphoid cell (ILC) family that lacks antigen receptors found in the classical T cells and B cells of the adaptive immune system [60]. They play a significant role in repairing damaged tissue and maintaining tissue homeostasis [61]. NK cells alter immune cell physiology either directly through receptor-ligand interactions or indirectly through cytokine secretion, direct contact-mediated lysis of autoaggressive T cells, and accelerated maturation of monocytes and dendritic cells. NK cells are essential in limiting cardiac viral infection and reducing cardiac eosinophilic infiltration in the mouse model of myocarditis [62]. NK cells are protective against the development of cardiac fibrosis both by directly limiting collagen formation in cardiac fibroblasts and by preventing the accumulation of specific inflammatory populations in the heart [62].

Multiple clinical studies have shown that coronary artery and ischemic heart disease patients have a decreased NK presence through either total numbers or phenotypic ability [62]. A chronic decrease in NK cells was correlated with low-grade cardiac inflammation, whereas patients that had restored circulating NK cells had little to no cardiac inflammation [62]. NK cell cytolytic impairment correlated with unstimulated levels of IL-6 in PBMCs of patients with heart failure. A decrease in NK cells was also observed in the coronary artery and ischemic heart disease [63].

NK cells prevent monocrotaline-induced endothelial damage [64, 65]. A study of fourteen patients with PAH (9 IPAH, 5 CTD) showed that deficiencies in NK cells might be associated with an increased risk of death in PAH patients [66]. NK cells from PAH patients showed functional impairment with a decrease in macrophage inflammatory protein 1b production and degranulation. Moreover, NK cells from PAH patients had higher levels of matrix metallopeptidase 9 and contribute to vascular remodeling [67]. These results indicated that NK cells have beneficial effects on the pathogenesis of PAH [62].

### 2.4. Platelets

Platelets play an essential role in cardiovascular disease both in the pathogenesis of atherosclerosis and in the development of acute thrombotic events [68]. Platelets modulate inflammatory response and produce proinflammatory mediators such as fibrosis-inducing PDGF and TGF-*β* antiangiogenic platelet factor 4 and B cell-activating CD40L [68, 69]. Occlusive intravascular platelet aggregates have been shown to cause ischemic myocardial damage both in the experimental animal and in patients [70]. Transgenic animals with decreased platelet aggregation were at reduced risk of coronary events. Risk factors for coronary artery disease, which include smoking, hypertension, and hypercholesterolemia, increase platelet hyperaggregability [71]. Patients with CHF have increased the risk of venous thromboembolism, stroke, and sudden death [72]. Thrombosis is the final pathogenic mechanism of acute ischemic events, including myocardial infarction, plaque rupture, and sudden cardiac arrest. Patients with acute myocardial infarction and unstable angina had increased platelet-derived thromboxane A2 and other prostaglandin metabolites [70]. CHF patients show increased whole blood aggregation and platelet-derived adhesion molecules as well as higher mean platelet volume. Although many studies have thus shown increased platelet activation in CHF, their role as inflammation-modulating cells is not well characterized [73]. A correlation between platelet-bound CD154 (CD40L) expression on platelets and serum levels of MCP-1 was observed [74]. Dual antiplatelet therapy (the cyclooxygenase inhibitor aspirin plus ADP receptor P2Y12 inhibitors) is the first-line treatment for STEMI.

Clinical studies show that patients with acute coronary syndromes have increased interactions between platelets and circulating leukocytes or neutrophils [58, 63]. Although not diagnostic, NLR and PLR were higher in HF patients than in age-sex-matched controls. PLR also was predictive of survivability after cardiac transplantation [75, 76]. Platelets have a significant role in the CTEPH type of pulmonary arterial hypertension [77]. In IPAH, thrombotic lesions and platelet dysfunction have been reported and may contribute to pathophysiology [36]. Abnormalities in the clotting cascade or platelets may contribute to thrombosis in pulmonary arteries. The von Willebrand factor, connected with endothelial dysfunction, plays a crucial role in platelet adhesion and aggregation in patients with IPAH [78].

### 2.5. Mast Cells

Mast cells are noncirculating immune cells that mature in target tissues in the presence of the c-kit ligand SCF from bone marrow-derived precursors [79]. Cardiac-resident mast cells increase in number in some disease conditions, including experimentally induced hypertension, myocardial infarction, and chronic cardiac volume overload, and promote cardiac remodeling and heart failure [79, 80]. They store and release a variety of active mediators, TNF-*α* and proteases such as tryptase, chymase, and stromelysin, implicated in the activation of MMPs, promoting fibrosis-stiffening-remodeling in cardiovascular disorders [42]. Inhibition of mast cell proteases was shown to prevent the development of cardiac fibrosis and improve LV dysfunction in experimental models of LV disease [70]. A role for mast cells in volume overload hypertrophy was established using c-kit-/- mice and drugs that prevent mast cell degranulation [71]. However, the role of mast cells was environment dependent. In homocysteine-induced cardiac fibrosis, mast cells had a protective role [81]. An increase in mast cell density was reported in the RV of the pulmonary banding model and chronic hypoxic rats. Mast cell density is also increased in the failing heart regardless of etiology. Patients with congestive heart failure with left ventricular assist devices had increased SCF and c-Kit gene expression and an increased number of mast cells after ventricular unloading [82]. It is found to contribute to atherosclerosis and plaque destabilization. Increased numbers of mast cells have been reported in explanted human hearts with dilated cardiomyopathy and heart failure [83].

### 2.6. Eosinophils

Cardiovascular manifestations of the hypereosinophilic syndrome are a common cause of morbidity and mortality in an otherwise uncommon disorder [84]. Pathological observations of the hearts of patients with prolonged eosinophilia have shown that both ventricles may be affected by thrombotic endocarditis associated with eosinophilic infiltration, vasculitis, and myocardial necrosis [84–86]. Causes of eosinophilia include drug-induced hypersensitivity reaction, infections such as HIV and helminth infections, systemic diseases such as rheumatoid arthritis, Crohn's disease, malignancies, and hypereosinophilic syndromes [86]. Binding of IgE antigen complexes and phagocytosis, release of granules with hydrolases, cationic and basic proteins toxic to the heart, and endothelial cells are capable of activating platelets by binding thrombomodulin [87]. In sustained eosinophilia, the accumulation of eosinophils in the interstitial compartment of the heart is deleterious to cardiac tissue and involves thrombus formation, thickening, and fibrosis of the endocardium [88]. There are some case reports of PAH in hypereosinophilic syndromes [89].

### 2.7. T Cells

Recent research has demonstrated that T lymphocyte-mediated immune response has a central role in CAD and the progression to heart failure [90–93]. In congestive heart failure, activation of the immune system leads to increased production and release of several proinflammatory cytokines. Recent studies have shown that alterations of adaptive immunity are critical for CHF pathophysiology [93]. In chronic ischemic cardiomyopathy, systemic expansion of CD4+ and CD8+ T cells and CD4+ Th1, Th2, Th17, and Treg subsets is found in the failing heart, circulation, and lymphoid organs. Mice deficient in Rag2, not having functional B and T cells, were protected from the transition from hypertrophy to heart failure after transverse aortic constriction (TAC) [92]. TCR*α*-deficient mice or mice with T cells depleted by anti-CD3 antibodies had a preserved cardiac function after TAC [94]. Th1- and Th17-polarized T cells have been reported to induce cardiac fibrosis and adverse cardiac remodeling [95]. Various studies have shown a central role for T cells in the progression of cardiac remodeling after myocardial infarction, in atherosclerotic plaques in mice. Autoreactive T helper cells with specificity for an antigen expressed in cardiomyocytes can promote the progression from hypertrophy to heart failure in response to pressure overload [91, 92]. After myocardial infarction, CD4+ T cells were reported to stimulate collagen matrix formation and thereby improve wound healing and survival by reducing the risk of myocardial rupture [92].

CD4/CD28 null, a unique subset of CD4 cells, is present in low frequencies in healthy individuals and increased in patients with chronic inflammatory diseases such as autoimmunity. These cells were increased in coronary heart disease [96]. CD4/CD28-null T cells accumulate preferentially in unstable ruptured coronary plaques and have been suggested to promote plaque instability and predispose ACS patients to recurrent acute coronary events (myocardial infarction) and indicate a poor prognosis [97, 98]. Patients with CHF show increased frequencies of proinflammatory CD4+ T helper 1 (Th1) and Th17 cells and lower frequencies of regulatory T cells, and these features correlate with disease severity [99]. Reduction of T cell infiltration may thus be a novel translational target in HF. CD8+ lymphocyte depletion is independently associated with death, decreasing 6 min walk distance and increasing NYHA classification [100]. CD8+ T lymphocyte depletion is present in some PAH patients and develops as the disease process deteriorates [101, 102]. Treg cell deficiency is associated with the progression of PAH [65, 102].

### 2.8. B Cells

Recent experimental and clinical observations suggest a link between activation of humoral immune responses after myocardial heart failure [103]. Animal studies using RAG2-/- SCID mouse models with defective T and B cells demonstrate that pathways leading to the activation of B cells are important players in heart failure and disease progression. Mice lacking programmed cell death protein-1 (PD-1-/-), a key factor for B cell differentiation, develop a severe form of spontaneous dilated cardiomyopathy [104]. High levels of circulating IgG bind specifically to cardiac myocytes. An interaction of B cells with T helper (Th1) cells stimulates cytokine production that can affect contractility as well as adverse remodeling [105]. Activated B cells can cause apoptosis of myocytes by activation of complement-mediated cytotoxicity [106]. In cases of multiple episodes of myocardial infarction where the immune system encounters myocardial proteins such as troponin, the memory B cell response may lead to a persistent inflammatory state, enhancing myocardial cell death and injury [107, 108]. In mouse models of ischemic CMP, the expression of cytokines, IgM, and IgG was increased 3-fold in the post-ischemic state compared to controls [109]. Beta-1 adrenergic receptor autoantibodies can induce apoptosis in isolated myocytes and exert a similar effect in vivo, causing myocardial dysfunction [110, 111]. Antibodies against the Na+/K+-ATPase also have been demonstrated [112]. Their presence seems to contribute to electrical instability in the heart, possibly making it prone to arrhythmias. This adverse effect may be caused by binding of the antibody to the alpha subunit of the Na+/K+-ATPase. Finally, antibodies specifically targeting the Kv channel interacting protein (KChIP) also are associated with dilated CMP and can potentially cause cardiomyocyte death as shown in a rat model [113].

A role for B cells in the progression of HF in humans is indicated by the presence of immunoglobulin, IgG3, with an equal proportion among ischemic and nonischemic patients. Activated complement components are increased in the circulation of patients with advanced disease and, more importantly, present in the failing myocardium [114]. In humans with dilated CMP and ischemic heart disease, antibodies reported in the literature include anti-muscarinic receptor 2, anti-mitochondrial M7, anti-actin, and anti-HSP-60 [103, 106]. In heart failure, HSP-60 present in the mitochondrial matrix will undergo translocation to the plasma membrane, where antibodies will bind and cause increased rates of apoptosis [100]. Limited clinical observations suggest that strategies to remove antibodies may have an impact on the course of HF. Taken together, evidence suggests that after myocardial injury, B cell activation triggers downstream effects that result in anticardiac antibody formation, complement deposition, and further myocardial injury.

## 3. Conclusion

Despite advances in therapies in heart failure, the prognosis of patients is poor. The research focused on understanding the role of the immune system in CHF has shown that both innate and adaptive immune responses are activated in the heart both in ischemic and in hypertrophic cardiomyopathy mice. However, their pathophysiological roles in heart failure are poorly understood. It is still not clear whether circulating levels of inflammatory mediators like TNF-*α* in patients with CHF are secondary response to myocyte injury. Investigations with anakinra (anti-IL-1 signaling), as well as the recently published CANTOS trial, support a role for subclinical inflammation in the progression of atherosclerosis and atherosclerotic-related diseases such as CHF [115]. Beneficial treatments for heart failure with drugs like ACEI and beta-blockers decrease monocyte function in animal models [57, 116]. However, clinical trials in patients with CHF using immunomodulatory therapy had poor outcomes. This could be due to the limitation of translating observations in animal models to humans, therefore warranting more studies in human tissues. Markers, such as total neutrophil count and NLR, can be evaluated more routinely in the clinical setting and correlated with parameters of cardiac function [26]. Studies are needed to understand how cardiac dysfunction activates the immune system. Tissue damage in the heart could cause activation of damage-associated molecular patterns (DAMPs) by pattern recognition receptors (PRRs) resulting in the activation of proinflammatory mediators for tissue repair. Tissue damage can also release GPCR-purinergic receptor agonists, ATP and ADP, which are DAMPs, leading to sterile inflammation response, regulating monocytes, macrophages, and T and B cells among others [117–119]. Finally, additional investigations are needed to better understand the role of immune cells in the heart in homeostasis and CHF to better define the therapeutic strategies targeting inflammation in patients with various forms of HF. Understanding their role in myocardial function and damage may be important not only in heart failure but also in preventing cardiac damage in patients undergoing immune-targeted therapies for cancer.

## Supplementary Materials

Supplementary Materials Abstract summary: role of inflammatory cells in heart failure.
