# Supplementary material for: Role of Inflammatory Cell Subtypes in Heart Failure
Source: J Immunol Res. 2019 Sep 2;2019:2164017. doi: 10.1155/2019/2164017 (PMC6745095; doi:10.1155/2019/2164017)
Supplement: Supplementary Materials — Abstract summary: role of inflammatory cells in heart failure. [file 2164017.f1.pdf]

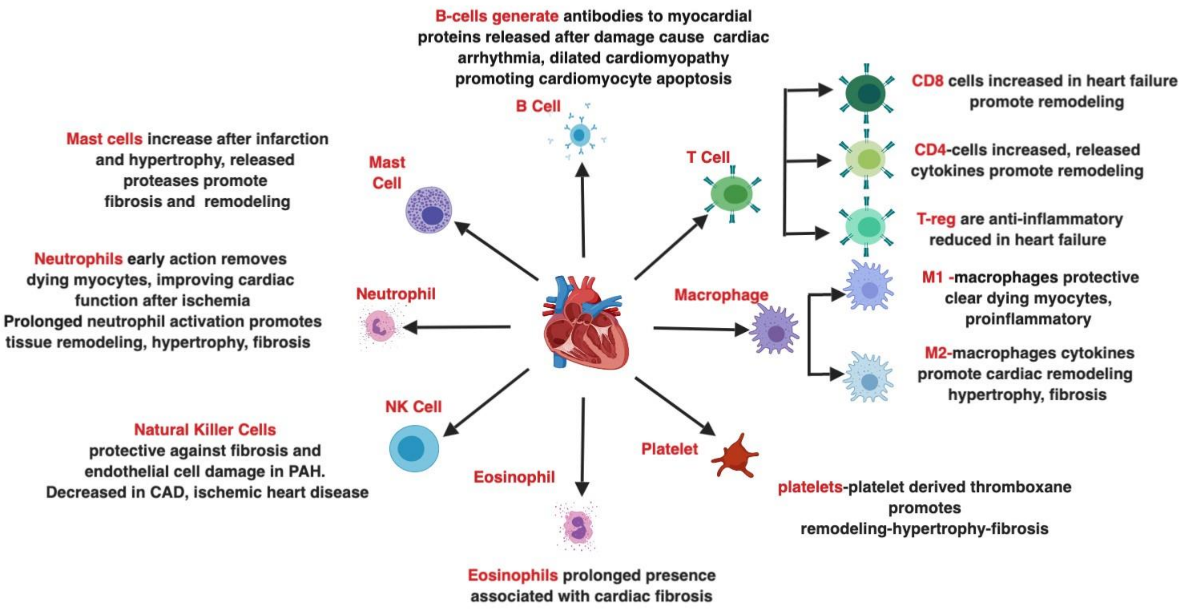

Abstract summary: Role of Inflammatory Cells in Heart Failure: Figure sum
inflammatory cells to heart failure
